# Genomic Analysis of QTLs and Genes Altering Natural Variation in Stochastic Noise

**DOI:** 10.1371/journal.pgen.1002295

**Published:** 2011-09-29

**Authors:** Jose M. Jimenez-Gomez, Jason A. Corwin, Bindu Joseph, Julin N. Maloof, Daniel J. Kliebenstein

**Affiliations:** 1 Department of Plant Biology, University of California Davis, Davis, California, United States of America; 2 Department of Plant Sciences, University of California Davis, Davis, California, United States of America; Georgia Institute of Technology, United States of America

## Abstract

Quantitative genetic analysis has long been used to study how natural variation of genotype can influence an organism's phenotype. While most studies have focused on genetic determinants of phenotypic average, it is rapidly becoming understood that stochastic noise is genetically determined. However, it is not known how many traits display genetic control of stochastic noise nor how broadly these stochastic loci are distributed within the genome. Understanding these questions is critical to our understanding of quantitative traits and how they relate to the underlying causal loci, especially since stochastic noise may be directly influenced by underlying changes in the wiring of regulatory networks. We identified QTLs controlling natural variation in stochastic noise of glucosinolates, plant defense metabolites, as well as QTLs for stochastic noise of related transcripts. These loci included stochastic noise QTLs unique for either transcript or metabolite variation. Validation of these loci showed that genetic polymorphism within the regulatory network alters stochastic noise independent of effects on corresponding average levels. We examined this phenomenon more globally, using transcriptomic datasets, and found that the Arabidopsis transcriptome exhibits significant, heritable differences in stochastic noise. Further analysis allowed us to identify QTLs that control genomic stochastic noise. Some genomic QTL were in common with those altering average transcript abundance, while others were unique to stochastic noise. Using a single isogenic population, we confirmed that natural variation at *ELF3* alters stochastic noise in the circadian clock and metabolism. Since polymorphisms controlling stochastic noise in genomic phenotypes exist within wild germplasm for naturally selected phenotypes, this suggests that analysis of Arabidopsis evolution should account for genetic control of stochastic variance and average phenotypes. It remains to be determined if natural genetic variation controlling stochasticity is equally distributed across the genomes of other multi-cellular eukaryotes.

## Author Summary

Understanding how genetic variation controls phenotypic variation is a fundamental goal of biology in both modern medicine and agriculture. Yet, frequently, even a large set of genetic polymorphisms do not fully explain variance of a phenotype within a discrete set of individuals. Numerous mechanistic theories have been proposed, e.g. epigenetics, but we postulated that there may be genome-wide polymorphism controlling phenotype stochastic noise among genotypes. This is similar to what is being found in studies of bet-hedging theory in prokaryotic or single-celled organisms or stability in eukaryotes. Utilizing Arabidopsis, we tested this hypothesis at a genomic level by mapping quantitative trait loci for stochastic noise in global transcriptomics, plant defense metabolism, circadian clock oscillation, and flowering time within a single non-stressful environment. We cloned and validated a set of genes including transcription factors and enzymes that control natural variation in phenotypic noise. These genes provided evidence that stochastic noise can vary independently of average phenotypes. Since population genetic models and quantitative genetic studies focus on natural genetic variations impact upon average phenotypes, these observations suggest that stochastic noise needs to be incorporated to better explain the genotype-to-phenotype link.

## Introduction

Almost all phenotypes are not fixed within species but instead exhibit significant levels of variation among individuals that is controlled by quantitative genetic loci. The study of such quantitative genetic variation has long been fundamental to evolution and ecology and is rapidly becoming a central focus of numerous other research fields, including breeding for improved crops and individualized medicine for humans. An ultimate goal of research on the basis of quantitative genetic variation is to generate a sufficient level of understanding to be able to predict phenotypic range of a species based on knowledge of that species' genetic variation. These efforts are complicated because phenotypic diversity is typically under polygenic control and can involve complex interactions with numerous factors including, but not limited to, the environment, development, epistatic interactions between genes, and potential higher-order interaction among these factors [1], [2]. Yet even in systems where these are understood to a significant degree, it has been difficult to develop predictive frameworks linking genotype to phenotype. Some of this difficulty has been ascribed to concepts such as epigenetic variance and difficulties in detecting small-effect loci [3], [4]. In this report, we propose that an additional explanation is the presence of numerous polymorphic loci that specify the amount of stochastic noise. If these polymorphisms are frequent in number, heritable and discrete from loci altering mean phenotpyes they can lead to an inability to fully describe the variance within any phenotype using current statistical approaches that focus solely upon the mean phenotype.

The idea that phenotypic variance is genetically determined is supported by a significant amount of research on how cells can limit stochastic noise/variance in genetic, metabolic, and signaling networks through network topology, a characteristic that is known as network robustness [5]–[10]. The specific topology of a network can increase or decrease the robustness of the output, wherein robustness is defined as the inverse of variance. Therefore, the genetic variation for loci within these networks could lead to allele specific changes in robustness/variance of the phenotype. Typically, robustness is thought to be under directional selection pressure to reduce the variance of an output and correspondingly increase network robustness. In evolutionary theory, this is predominantly described as canalization wherein genes function to minimize the variance (maximize the robustness) of a phenotype [11], [12]. In yeast, phenotypic and genetic robustness (i.e. canalization) were shown to correlate using genomic knockout datasets [13]. In plants, loci that control natural variation in canalization of critical developmental processes such as cotyledon opening and leaf formation have been mapped and cloned revealing that canalization genes can be known members of regulatory networks controlling these processes [14], [15]. Additionally, it has been shown that heat-shock protein 90 plays a major role in canalizing existing natural variation possibly as a pool of hidden evolutionary potential [16]-[18]. However it should be noted that the genomic level of, distribution of and importance of naturally variable loci controlling within-genotype variance is currently not fully described in most eukaryotes [19].

While canalization and robustness research focuses on the benefits of decreasing within-genotype variance, there is evidence that increases in per genotype variance can also be beneficial. This is occasionally called the portfolio effect wherein the fitness of a genotype is determined by the portfolio of phenotypes that it can obtain [20]. In some bacterial settings rapid environmental fluctuations have been shown to favor the development of stochastic switching as the optimal means of response [21]–[25]. Similarly in eukaryotes, it has been shown that natural variation can alter stochastic noise of gene expression [19], [26] and that stochastic noise in defense phenotypes could help to delay the evolution of counter-resistance in biotic pests [27], [28]. As such, it is possible that there is wide-spread genetic variation controlling stochastic noise in eukaryotic phenotypes that may play a beneficial role in the evolution of these organisms [16]–[18], [25]. However, little is understood about the genomic distribution of natural quantitative genetic variation for stochastic noise in eukaryotes or about the direction of selection on that natural variation.

The concept that stochastic noise is genetically determined in a quantitative, polygenic manner is supported by the analysis of stochastic variation in expression of a *MET17* reporter fusion construct in *Saccharomyces cerevisiae* [29]. This study identified significant genetic diversity regulating stochastic noise of gene expression and showed that stochastic noise was a complex trait controlled by at least three quantitative trait loci (QTLs) [29]. However, given the nature of these alleles, it is not known if these polymorphisms are present in wild populations or are laboratory derived. Additional evidence comes from the study of the *S. cerevisiae* galactose regulon where it was found that genetic manipulation of the regulatory feedback loop could lead to increased stochastic noise in the network's output [30], [31]. Genetic control of stochastic noise has also been identified using QTLs for yield stability in crops [32] and gene expression in 18 isogenic mouse lines [19]. Further, it has been shown that HSP90 likely buffers genetic variation which could appear as stochastic noise in fluctuating environments, but little is known about the genomic distribution of natural variation in stochastic noise within a constrained environment [16]–[18]. These studies indicate that there is the genetic variation to regulate stochastic noise in physiology and gene expression suggesting that stochastic noise itself is a phenotype subject to natural selection with potential for pressure in both positive and negative directions.

To begin testing the genomic extent of natural genetic variation in stochastic noise we used the model plant, *Arabidopsis thaliana*. Arabidopsis is quickly becoming a key organism in the study of complex traits through the use of systems biology and quantitative genomics approaches [33]–[40]. This is due to large repositories of transcriptomic and metabolomic data for homozygous QTL and association mapping populations that, when combined with whole genome sequence of natural accessions, provides the ability to rapidly develop and test hypotheses as well as find causal genes underlying specific loci of interest [41]–[44]. This has enabled the identification and validation of numerous genes and defense pathways under natural selection [45]–[50]. Among these defense mechanisms with known selective consequences are the glucosinolate metabolites, thioglucosides that provide defense against numerous biotic pests and whose accumulation is heritable and under balancing or fluctuating selection in the field [51]–[62]. This makes Arabidopsis an ideal system to search for the genetic and molecular basis of complex phenotypes, such as stochastic noise, in higher organisms.

Using previous datasets, we identified QTLs that control natural variation in stochastic noise of glucosinolate metabolites and related transcripts within a single controlled environment. There were QTLs unique for the different phenotypic levels and we showed that known genes underlying these glucosinolate loci led to altered glucosinolate stochastic noise. We then extended this analysis to show that the Arabidopsis transcriptome shows significantly heritable stochastic noise for expression levels. Further, we were able to identify QTLs that control global stochastic noise in gene expression. Some loci were in common with those altering the average transcript abundance while others appeared unique to controlling transcriptomic stochastic noise. Using an existing single isogenic population, we confirmed that natural variation at the *ELF3* locus alters stochastic noise in both physiological and metabolic phenotypes. Given the wide spread genomic variation controlling natural variation in stochastic noise in a single environment that we found within the wild Arabidopsis germplasm, our results suggest that any analysis of Arabidopsis evolution needs to account not only for genetic control of average phenotype value but also for genetic control of stochasticity. It remains to be determined how widely distributed this level of genomic natural variation exists for stochasticity within a wider range of multi-cellular eukaryotes.

## Results

### QTLs controlling stochastic noise of plant defense metabolism

To test if there is genetic variation affecting stochastic noise in the higher plant *Arabidopsis thaliana* we used a previous analysis of quantitative variation in glucosinolate defense metabolites [63]. The glucosinolate biosynthetic, transport and regulatory networks have been highly characterized [64]–[69], providing extensive information about the loci responsible for differences in mean glucosinolates within *Arabidopsis thaliana* accessions [60], [70]–[72]. Given the extensive knowledge it is possible to use existing glucosinolate data to search for QTLs controlling stochastic noise in glucosinolate accumulation. If stochastic noise QTLs are found they can be compared to existing analyses to determine if the same QTLs control phenotypic mean.

**Figure 1:**
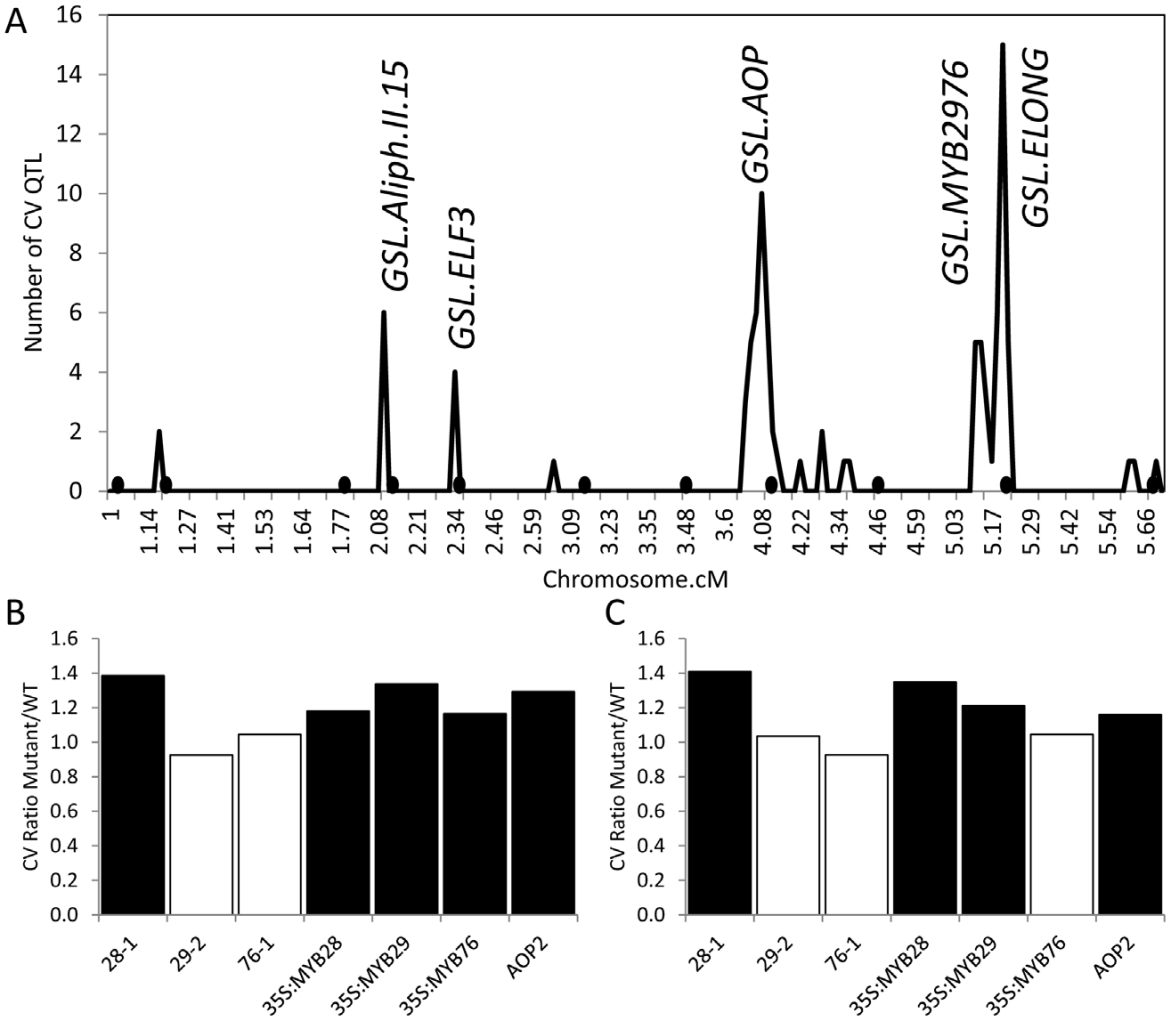
QTLs and known genes controlling per line CV in Glucosinolates. (A) Shows the position of QTLs for 40 CV glucosinolate phenotypes. Random permutation threshold for significant enrichment of co-localized QTL is 2. The y-axis indicates the total number of phenotypes controlled by a given QTL. Labels show the position of known QTLs and the new *MTB2976* QTL. The 392 line Bay x Sha RIL population was utilized to map QTL for this analysis and this has a smaller genetic map than the 211 RIL subset due to a lower marker density. Black circles below the x-axis show the position of QTLS found to control the average phenotype [63]. *GSL.ELF3* was previously described as *GSL.ALIPH.II.42* [63]. (B) The effect of single gene variation at *GSL.AOP* and *GSL.MYB* QTLs on the CV for aliphatic glucosinolates is presented as the ratio of the average aliphatic glucosinolate CV within the single gene variant to the parental WT Col-0. 28-1, 29-1 and 76-1 are homozygous insertional T-DNA mutants for *MYB28*, *MYB29* and *MYB76* respectively. The *AOP2* genotype is the Arabidopsis Col-0 accession (contains a natural knockout in *AOP2*) expressing a functional *AOP2* enzyme [115]. Black boxes show those comparisons where the single gene variant was significantly different from WT (P<0.05, Levene's F-test comparing variance between mutant and WT genotypes). (C) The effect of single gene variation at *GSL.AOP* and *GSL.MYB* QTLs on the CV for indolic glucosinolates is presented as the ratio of the average aliphatic glucosinolate CV within the single gene variant to the parental WT Col-0. 28-1, 29-1 and 76-1 are homozygous insertional T-DNA mutants for *MYB28*, *MYB29* and *MYB76* respectively. The *AOP2* genotype is the Arabidopsis Col-0 accession (contains a natural knockout in *AOP2*) expressing a functional *AOP2* enzyme. Black boxes show those comparisons where the single gene variant was significantly different from WT (P<0.05, Levene's F-test comparing variance between mutant and WT genotypes).

A previous analysis of glucosinolate variation in the *Arabidopsis thaliana* recombinant inbred line population (RIL) derived from the Bayreuth (Bay) and Shahdara (Sha; syn:Shakdara) accessions [42] reported both the mean glucosinolate accumulation and standard deviation per line for three replicated experiments quantifying concentrations of 62 different glucosinolate phenotypes in 392 RILs [63]. We used this information to obtain the coefficient of variation (CV) of each glucosinolate phenotype for each RIL by dividing the standard deviation of the phenotype by its mean, and used this dimensionless measure of stochastic noise in glucosinolate accumulation to perform QTL analysis [21], [26]. This identified five QTL hotspots controlling differences in glucosinolate CV (Figure 1). The pattern of CV QTL was similar to that found for QTL affecting differences in mean phenotype where *GSL.ELONG* and *GSL.AOP* are the major loci followed by two additional hotspots on chromosome 2 that had also been found to affect mean glucosinolates but were less significant than for glucosinolate CV (Figure 1) [63]. Further, we found a new QTL for CV that was not found for the mean phenotype within this population but had previously been found as a glucosinolate QTL in other populations, *GSL.MYB2976* (Figure 1) [63]. There were also several QTLs that affected the mean phenotype but did not cause significant differences in glucosinolate CV (Figure 1) [63]. Thus, it is possible to find QTLs controlling CV differences and these are not necessarily the same loci as those that affect the phenotypic mean.

Fortunately, several of the identified QTLs have already been cloned and previously published single gene validation lines exist with published glucosinolate data to allow rapid validation of the CV phenotypes [63], [67], [73]. We have previously shown that the *GSL.AOP* locus is controlled by differential expression of two enzymes, *AOP2* and *AOP3*, which evolved from a tandem duplication event to control different reactions with the same precursor [63], [74]. Using the same data set which previously showed that the QTL allele for increased glucosinolate accumulation and glucosinolate network transcript abundance was caused by expression of the *AOP2* gene [63], we showed that introducing the *AOP2* gene into a natural knockout background (Col-0) also significantly increased glucosinolate CV (Figure 1B and C). This increase correlates with the elevated CV found in Sha, which contains the functional *AOP2* allele at the *GSL.AOP* locus (data not shown).

The *GSL.MYB2976* locus co-localizes with a previously cloned QTL from a different RIL population (L*er* x Cvi) that is controlled by two glucosinolate transcription factors, *MYB29* and *MYB76* [67], [72], [73]. We used data from previous single gene manipulations of *MYB29* and *MYB76* as well as the related *MYB28*, also linked to glucosinolate QTLs in other populations, to test if these genes can influence natural variation in glucosinolate CV [62], [67]–[69], [73], [75]. Interestingly, increasing or decreasing *MYB28* expression significantly increases CV for all glucosinolates (Figure 1B and C). This is in contrast to previously published data showing that increasing MYB28 expression increased glucosinolate content while decreasing MYB28 expression correspondingly diminished glucosinolate content. Together this suggests that the effect of genetic variants on CV and mean is not always correlated [67]–[69], [73], [75].

In contrast to *MYB28*, only increases in *MYB29* and *MYB76* expression altered metabolite CV while decreased expression at either gene had no impact on glucosinolate CV (Figure 1B and C). This differs from their impact on mean glucosinolate accumulation where increases and decreases in all three gene expression lead to correlated increases and decreases in glucosinolate metabolites [67]–[69], [73], [75]. Interestingly, the natural variation in gene expression of MYB29 and 76 in the Bay-0 x Sha population appears to be a shift from a Col-0 like level in the Bay-0 genotype to elevated expression in the Sha genotype [40], [76] agreeing with the observed introduction of a CV QTL in this position. It is possible absence of a *MYB2976* QTL altering the mean phenotypes may be an issue of not having sufficient RILs to identify this locus in the background of the other QTLs showing significant epistatic interactions [63]. To test if the use of CV may be biasing our analysis, we used Levene's F-test to compare variances between the various mutants and WT and obtained similar results (Figure 1). In summary, *MYB28*, *MYB29*, *MYB76* and *AOP2* alter glucosinolate CV, mean and unadjusted variance (Figure 1) [63], [67]–[69], [73], [75]. Since *MYB29*, *MYB76* and *AOP2* underlie CV QTLs, they are good candidates to control natural variation in glucosinolate CV within *Arabidopsis thaliana*. The observation that *MYB28* and *MYB29* perturbations have similar consequences upon mean glucosinolate accumulation but different influences on glucosinolate CV shows that the CV is not being driven by underlying changes in mean and is a valid approach for this analysis.

### QTLs controlling stochastic noise of plant defense transcript abundance

The *GSL.AOP* and *GSL.MYB2976* QTLs control differences in both the mean accumulation of glucosinolate metabolites and the relevant biochemical pathway transcripts [63], [67], [73]. Having found that these QTL controlled differences in CV for glucosinolate metabolites, we next tested whether these QTL also control differences in CV for transcripts involved in glucosinolate production. We used pre-existing microarray data [40], [77] and found little evidence for impacts of the *GSL.AOP* and *GSL.MYB2976* loci on the CV of transcript accumulation for individual transcripts in the GLS pathway (Figure S1), in contrast to their effect on CV for glucosinolate metabolites. Hereafter these loci are referred to as CV eQTL (CV eQTL  =  a QTL altering the coefficient of variation in transcript accumulation) to delineate them from standard eQTL (eQTL  =  a QTL altering the mean transcript accumulation). Similarly, there was no evidence that these loci impact the GLS related biosynthetic networks (Figure S2). This is in contrast to previous observations showing that *AOP2, MYB29* and MYB76 can cause changes in glucosinolate pathway transcription and are known eQTL (expression QTL) hotspots for mean glucosinolate transcript abundance[63]. This is not entirely surprising as glucosinolate regulation shows extensive hallmarks of incoherent feed-forward loops [65], [67], [73] which can cause non-linear relationships in variance at different output levels. Thus the difference in CV partitioning between metabolites and transcripts at these loci is not entirely unexpected. Together, these data suggest that although the *GSL.AOP* and *GSL.MYB2976* QTLs and the underlying causal loci (*AOP2*, *MYB29* and *MYB76*) affect the mean transcript and metabolite abundance in the GLS pathway, and the CV of metabolite abundance, they don't alter the CV of transcript accumulation in this pathway. Interestingly, a hotspot on chromosome 2, controls the per transcript CV abundance of most genes in the GLS pathway and CV in glucosinolate content (*GSL.ELF3*, Figure 1). This locus fits the definition of a network CV eQTL as it alters the CV of the glucosinolate transcript network.

While there was no network CV eQTL at the *GSL.AOP* locus, the *AOP2* and *AOP3* genes showed evidence for a *cis* positioned eQTL controlling the CV for transcript accumulation for only these two genes and not the entire pathway (Figures S1 and S2). Interestingly, not all glucosinolate associated transcripts known to have a large effect *cis*-eQTL also had a *cis*-CV eQTL. For instance, the *GSL.MAM* locus contains cis-eQTL for the *MAM* genes yet there was no corresponding cis-CV eQTL (Figure S1) [63]. If our use of CV was solely tracking changes in mean abundance, the large effect *cis*-eQTL by default should have large effect *cis*-CV eQTL. The lack of this absolute relationship suggests that changes in mean are not driving changes in CV and supports the use of CV for mapping stochastic noise QTLs. Additionally supporting this is the fact that we utilized the same threshold estimation approaches for both *CV*-eQTL and *cis*-eQTL detection arguing against this being different statistical power issues [40].

### Analysis of quantitative genetics controlling stochastic noise in gene expression

**Figure 2:**
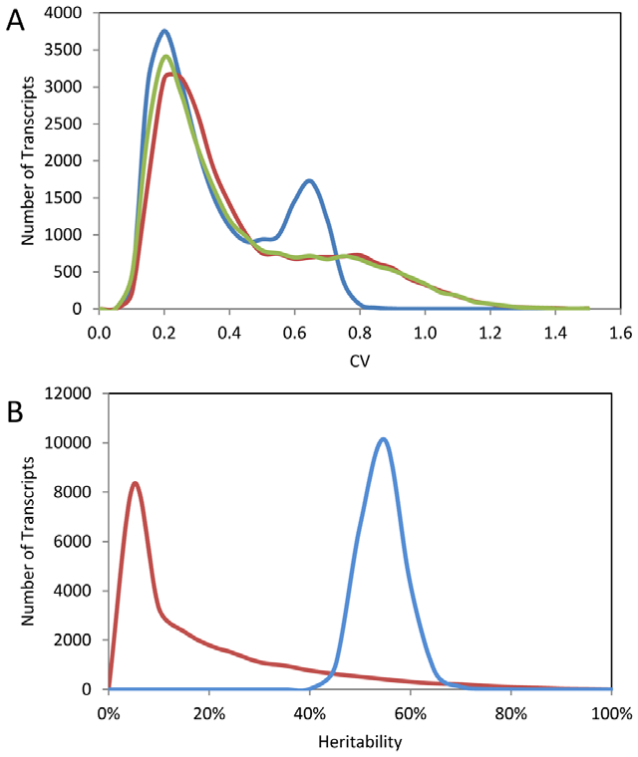
Summary of per transcript CV in Bay, Sha, and 211 RILs. The average per transcript CV for 22,746 transcripts from Bay, Sha and across 211 Bay x Sha RILs was measured from two independent microarray experiments each containing independent biological replicates per genotype. (A) The distribution of CV across the transcripts is shown for Sha (yellow-green), Bay (red) and 211 Bay x Sha RILs (blue). For the RIL histogram, CV is averaged across all 211 RILs per transcript. The Bay and Sha distributions are significantly different (t-test, *P*<0.001). (B) The estimated heritability for per transcript CV between the Bay/Sha parents (Red) and within the 211 RILs (Blue).

**Figure 3:**
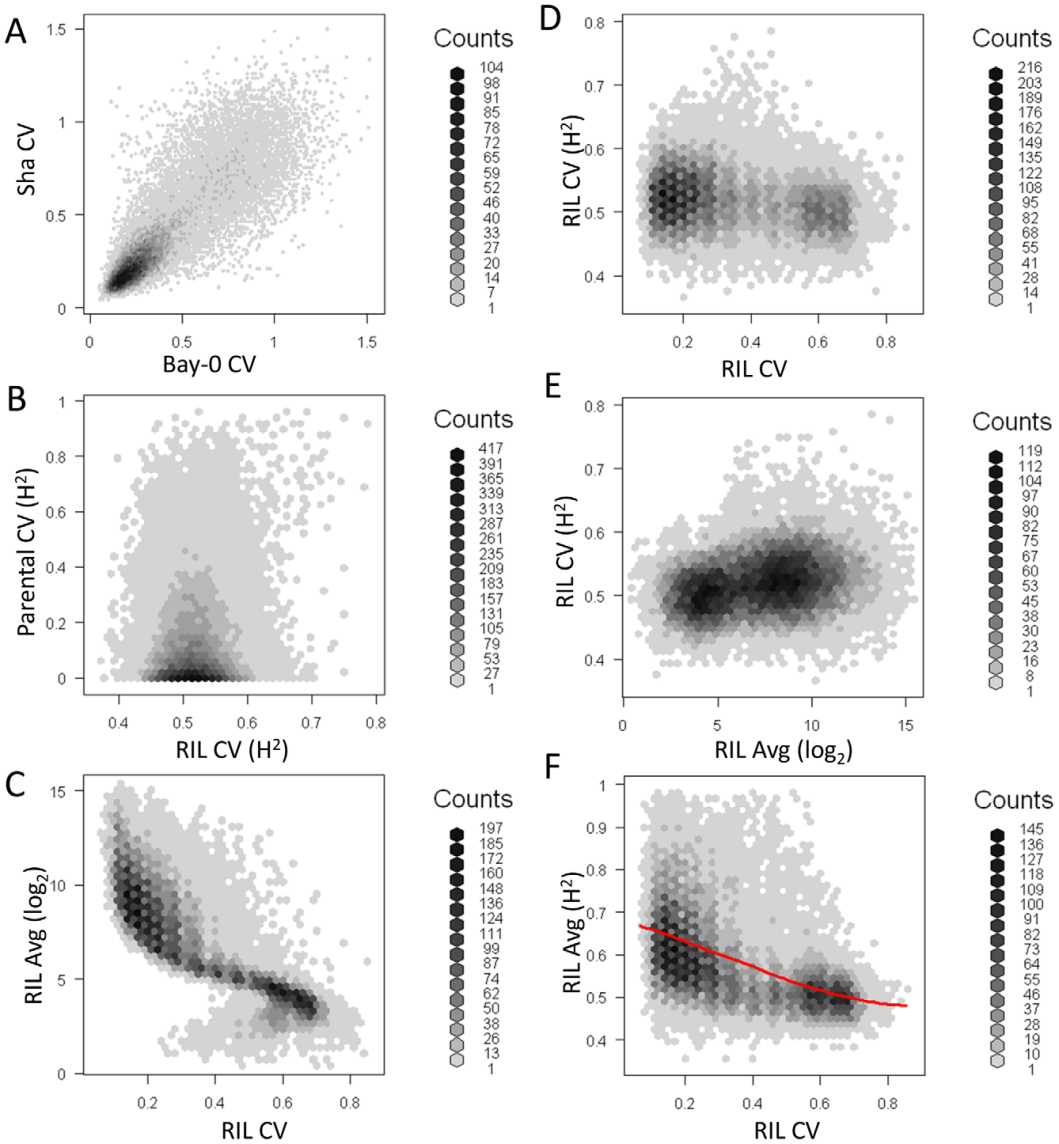
Relationship of transcript CV to heritability and average expression. Heritability, average expression and CV for Bay, Sha and the RILs was measured for all 22,746 transcripts. Graphs are hexbin histograms with the counts per hex shown to the right of each graph. (A) Correlation of measured transcript CV in Bay and Sha. (B) Comparison of transcript CV heritability in the Parents and RILs. (C) Comparison of average transcript expression and average transcript CV within the RILs. (D) Comparison of transcript CV heritability and average transcript CV within the RILs. (E) Comparison of transcript CV heritability and average transcript abundance within the RILs.

The above analysis of existing glucosinolate quantifications suggests that there is significant genetic control of the CV for these defense metabolites. The CV itself may be under selective pressure to generate differences in stochastic variability between different natural populations of Arabidopsis [25], [28]. To query if genetic control of phenotypic CV is a global phenomenon within Arabidopsis, we used a pre-existing dataset consisting of replicated microarray experiments conducted on 211 lines of the Bay x Sha RIL population and the RIL parents [40], [77]. The distribution of CV across the transcripts was similar between Bay and Sha with a statistically significant difference of Bay showing a slight shift of the peak towards a higher CV (Figure 2A). Interestingly, the distribution of CV across the transcripts was more distinctly bimodal within the RILs suggesting significant transgressive segregation in the population only impacting a specific subset of transcripts (Figure 2A). The replicated nature of this experiment allowed us to directly assess the heritability of per line CV differences in both the parents and the RILs across 22,746 different transcripts representing the majority of the genome. The per transcript CV were correlated between Bay and Sha with an average heritability of 17% (Figure 2B and Figure 3A). The average heritability of per transcript CV was much higher in the RILs than the parental genotypes with an average heritability of 57% (Figure 2B). This is similar to the average heritability reported for the mean transcript abundance for the same experiment (∼68%) with the majority of this difference being due to the lack of a high heritability tail for transcript CV as compared to heritability for mean transcript abundance [40], [77]. As found previously for the mean transcript values, there was very little relationship between the heritability as measured in the Bay/Sha parents versus the RIL (Figure 3B). For the mean transcript abundance, this discrepancy was explainable by transgressive segregation due to QTLs of opposing effect and is likely true for CV-eQTLs as well, suggesting that similar levels of robustness in the two parents are obtained via different genetic networks [40], [77]. Supporting this is the observation that the standard deviation of transcript CV across the RILs is significantly greater than would be expected by modeling the expected CV using the parental values. In 1000 models, the maximal standard deviation of CV averaged across the transcripts in the RILs was 0.09 with a mean of 0.08. In contrast, the actual biological values showed an average standard deviation of CV per transcript across the RILs of 0.17 indicating that the RILs show a significantly larger distribution of CVs per transcript per RIL than would be expected given the parental value.

One concern with CV and any other estimate of variance is the potential for a correlation between variance and mean. The above analysis with glucosinolate accumulation did not suggest that this was a concern within Arabidopsis natural variation because we could identify instances where there were QTLs with large effect on the mean but no effect on the CV, even when identical approaches were used to determine significance thresholds. Within the RIL transcriptomic data, we did observe a statistically negative correlation (P<0.001) whereby transcripts with the lowest average abundance had the highest CV and vice versa however this correlation explained only 0.8% of the total variation in CV leaving 99.2% of the variation to be available for genetic control of CV independent of the mean (Figure 3C). This significant but minimal negative correlation likely derives from technical issues in microarrays surrounding the detection of lower expressed transcripts using Affymetrix microarray technology. To test if this technical issue constrains our ability to identify biologically controlled transcript CV, we compared the average per transcript expression to the heritability of per transcript CV within the RILs. This analysis showed that higher expressed genes had only a slightly more reproducible transcript CVs, therefore the technical issues surrounding low expressed genes does not impact our ability to identify biologically controlled CV (Figure 3E). Additionally, the magnitude of per transcript CVs in the RILs showed very little relationship to the heritability of per transcript CV suggesting that any CV/expression level correlation is not creating relationships at higher levels (Figure 3D). Thus, the use of CV to map QTLs for the transcripts appears to be valid. Interestingly, there was a strong negative correlation between the heritability of transcript abundance and the transcript CV, such that transcripts with the lowest CV had the highest heritability (P<0.0001, R2 = 0.59; Figure 3F). To ensure that the relationship between mean transcript abundance and transcript CV was not driving this correlation we repeated the analysis as a partial correlation while controlling for mean transcript abundance, this still showed a highly significant negative relationship between the heritability of transcript abundance and the transcript CV (P<0.0001, R2 = 0.42). Together, this suggests that quantitative genetic control of CV is a genome wide phenomenon within *Arabidopsis thaliana* that is not limited to defense metabolites and is at least partially independent from genetic variation controlling the mean phenotype.

### Identifying CV eQTLs controlling stochastic noise for the Arabidopsis transcriptome

The high estimated heritability of per transcript CV within the Bay x Sha RIL population suggests that it is possible to map CV eQTL for all transcripts. We used composite interval mapping to map CV eQTL for all 22,746 transcripts within 211 lines of the Bay x Sha population previously used to map eQTL [40], [77]. This identified 98,014 significant CV eQTLs that altered the stochastic noise for 21,974 transcripts for an average of nearly 4 CV eQTL per transcript (Figure S3). This is nearly twice the number of eQTLs per transcript found using the average transcript abundance as a phenotype [40]. This difference may be due to the use of two different experiments in the CV eQTL analysis, whereas the eQTL analysis used just one experiment, reducing its statistical power [40]. Given that we used identical methods to identify global permutation thresholds for both datasets, we do not feel that a higher false positive rate can explain the elevated number of CV eQTLs [40], [78]-[80]. In addition, the elevated number of CV eQTLs is not universal as the glucosinolate transcript measurements actually identified more eQTLs than CV eQTL (Figure S1) [63]. Thus, the elevated CV eQTL level may be more indicative of the specific biological process within which that transcript functions.

**Figure 4:**
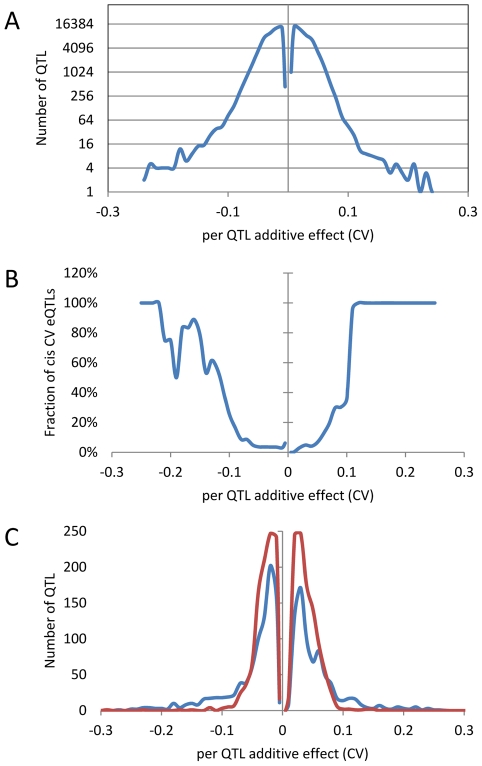
Distribution of additive effects of allele variation upon CV for significant CV eQTL. The absence of values near 0 is likely due to statistical power that does not allow detection of these loci if they exist. Additive effect is defined as the estimated impact of the Sha allele. (A) The distribution of all CV eQTL additive effects is shown in a log scale to allow for better visualization of the tails. (B) The fraction of CV eQTL that are due to *cis* localized CV eQTL. (C) Comparison of additive effect between genes with a *cis* eQTL for just transcript CV (Red) and for both transcript CV and mean (Blue).

**Figure 5:**
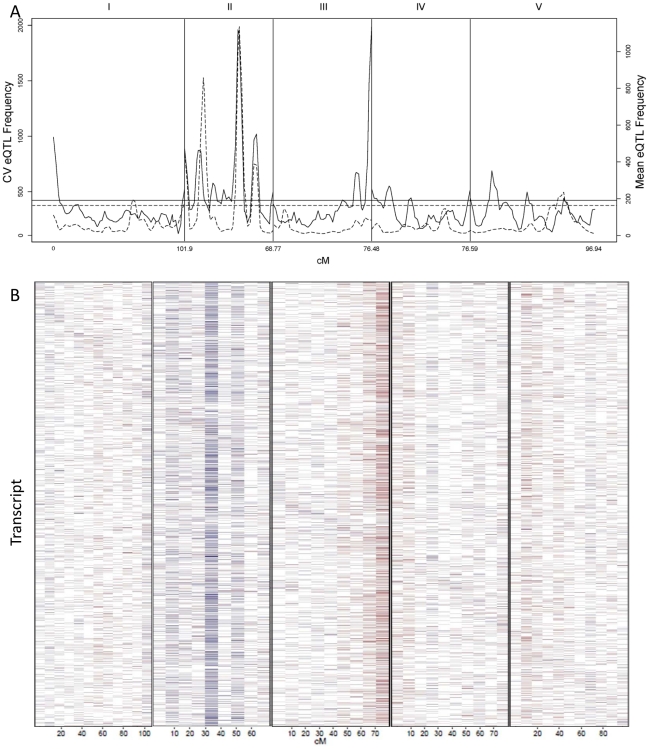
CV eQTL for Bay x Sha RIL population. CV eQTLs were mapped for 21,974 out of 22,746 transcript CVs tested. (A) The solid line shows a 2.5 cM sliding window analysis of the average number of CV eQTL per region. Chromosomes are labeled at the top and cM per chromosome is at the bottom. The horizontal line represents the α = 0.05 threshold for regions with a significant enrichment in CV eQTL (1,000 random permutations). The dashed line shows an equivalent 2.5 cM sliding window analysis of the average number of mean eQTL per region using the same transcripts as a reference. (B) Heat map of CV eQTL for transcripts. Chromosomes are as labeled in A. The physical position of the genes from which the transcript are derived are ordered on the y-axis by their physical position with the first gene on chromosome I at the top and the last gene on chromosome V on the bottom. Red represents a negative effect of the Bay allele upon CV while blue is a positive effect. Genes are plotted in bins so the map positions do not exactly align with those in A.

An analysis of the distribution of additive effects for the CV eQTL showed a slight bias towards Bay alleles having a negative impact on CV (50429 CV eQTLs with Bay additive effect <0 versus 47585 with Bay additive effect >0)(Figure 4A). The vast majority of CV eQTLs had absolute effects less than 0.1 CV and these were almost entirely acting in *trans* (Figure 4A and B). In contrast, CV eQTL with absolute effects greater than 0.1 were predominantly acting in *cis* (Figure 4B). This is similar to eQTL controlling the mean accumulation of a transcript where on average *trans*-eQTLs have smaller additive effects than *cis*-eQTLs [38], [40]. This analysis identified 3,720 transcripts as having a *cis*-CV eQTL, in contrast with the 5,127 transcripts having a *cis*-eQTL for mean expression level (Figure 4) [40]. While about ¼ of all eQTLs detected were *cis*, only 1/26^th^ of all CV eQTL were *cis*, showing that natural variation at *trans* positions is dramatically more prevalent in controlling transcript CV than average expression (Figure 4) [40]. As expected by the decreased ratio of *cis*-CV eQTL relative to that found for eQTL, the *cis* diagonal, while present, was very faint (Figure 5B). Only 1,660 transcripts had both a *cis*-eQTL and *cis-*CV eQTL and these included nearly all of the large effect CV eQTLs (Figure 4) [40]. Thus, while a *cis*-eQTL can be associated with a *cis*-CV eQTL, it is not a necessity (Figure 4). These results show that stochastic noise measured as CV in transcript abundance is a highly heritable trait suitable for genome wide QTL analysis in multi-cellular eukaryotes. As in eQTL analyses of mean transcript abundance, differences in the CV of transcript accumulation seem to be broadly caused by abundant loci acting in trans, while substantial changes are less frequent and usually associated with variation in cis.

### Identifying QTLs controlling global stochastic noise in transcripts

We counted the number of loci per chromosomal position controlling stochastic noise within the Arabidopsis transcriptome to better understand the genomic distribution of CV eQTLs (Figure 5A). This identified a number of locations within the genome that contain trans-hotspots for CV eQTL. Several of these were in common with eQTL *trans*-hotpots that had previously been identified such as the locations on Chromosome II. However, the relative impact of the trans-hotspots upon the transcriptome was different for the two traits (Figure 5A) [40]. For instance, the *trans*-hotspots at 12 and 42 cM on chromosome II caused similar numbers of eQTL, yet the hotspot at 42 cM affected many more CV eQTLs than the hotspot at 12cM. Additional hotspots were detected with CV eQTL that were not detected using mean transcript accumulation, most notable is the locus at the bottom of chromosome III that is the highest *trans*-hotspot for CV eQTL but barely registered for eQTL (Figure 5A) [40]. Two other apparent CV eQTL specific *trans*-hotspots were peaks over the permutation threshold near the *GSL.AOP* and *GSL.MYB2976* loci on chromosomes IV and V (Figure 5A) [40]. However, none of the glucosinolate transcripts' CVs were regulated by the *trans*-CV eQTL hotspots near *GSL.AOP* and *GSL.MYB2976* (Figure S1). This raises the question of whether these CV loci near *GSL.AOP* and *GSL.MYB2976* are due to pleiotropic consequences of the metabolic CV controlled by *GSL.AOP* and *GSL.MYB2976* (Figure 1) or if there are additional genes in these regions that alter transcriptomic CV.

The detected CV eQTL hotspots have additive effect biases, with most of the CV eQTLs in one hotspot increasing the CV in the same direction, as noticed before for eQTL hotspots (Figure 5B) [40]. The two major hotspots had opposite effects; with the Sha allele causing increased stochastic noise at the hotspot in chromosome III and decreasing stochastic noise in all hotspots on chromosome II (Figure 5B).  =  This observation further shows that increased mean abundance does not inherently cause increased CV. Thus, transcript mean abundance and CV are not measures of a single phenotype and instead can involve different genetic mechanisms even when investigating the same locus.

**Figure 6:**
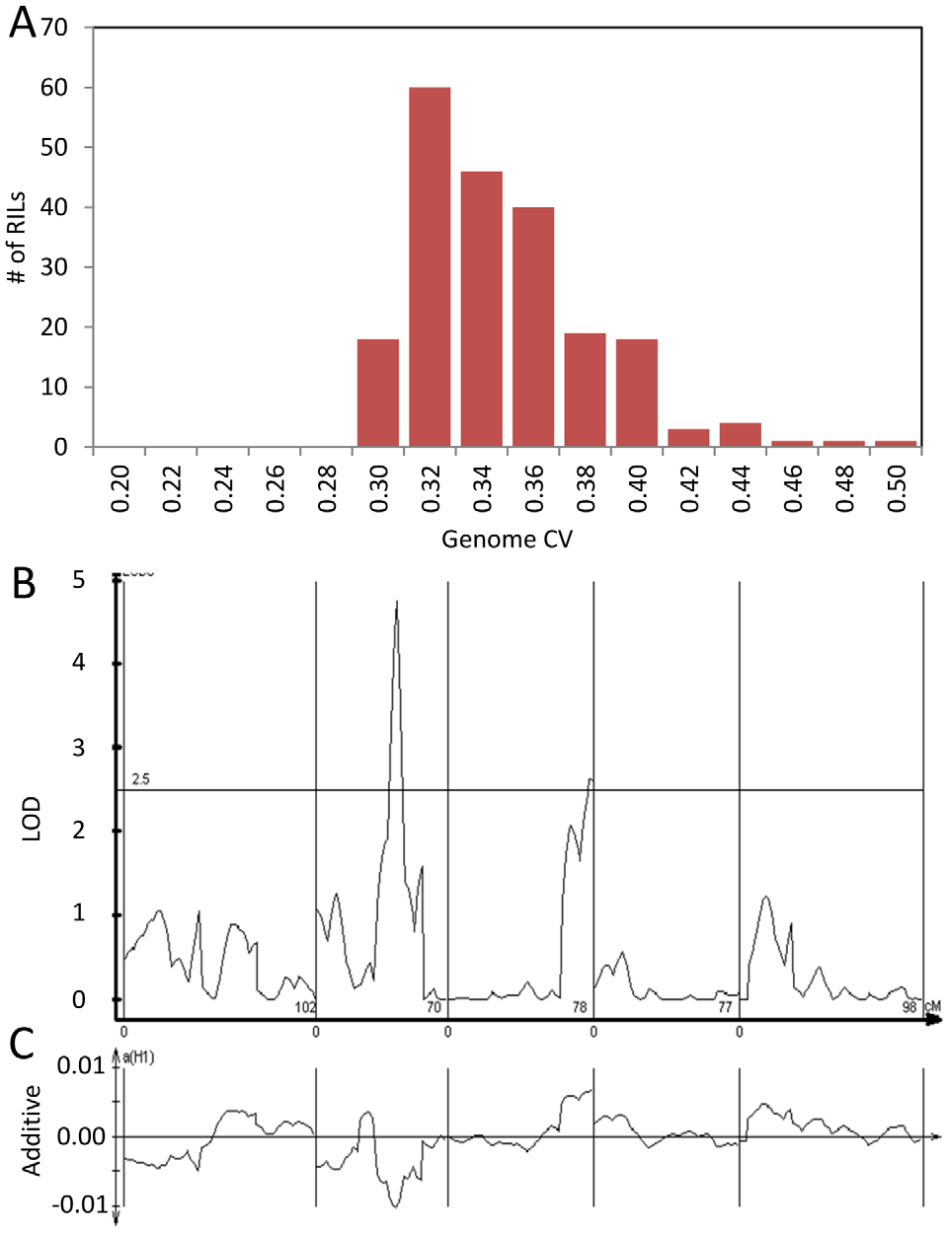
Genetics of global transcript CV. The average global transcript CV per RIL was taken by averaging across all 22,746 transcript CVs per RIL. The Bay and Sha parents global transcript CV are respectively 0.411 and 0.399 (*P*<0.05, ANOVA). Bay and Sha parental plants were grown with the RILs. (A) Histogram of global transcript CV across the 211 RILs. (B) The LOD plot for mapping QTL within the 211 RILs that control global transcript CV variation. (C) Additive effect plot for the QTL. The direction is based on the Sha allele.

The global effect of *trans*-CV eQTL hotspots led us to test if we could directly map QTL controlling genome-wide transcriptomic CV (as opposed to per transcript CV). Taking the average CV across all transcripts showed that Bay and Sha have different CV and that the main source of this is the previously identified loci on Chromosome II and III (Figure 6). Thus, these loci appear to have genome wide effects upon stochastic noise of gene expression and likely other traits.

### *ELF3* genetic variation controls global stochastic noise in numerous phenotypes

**Figure 7:**
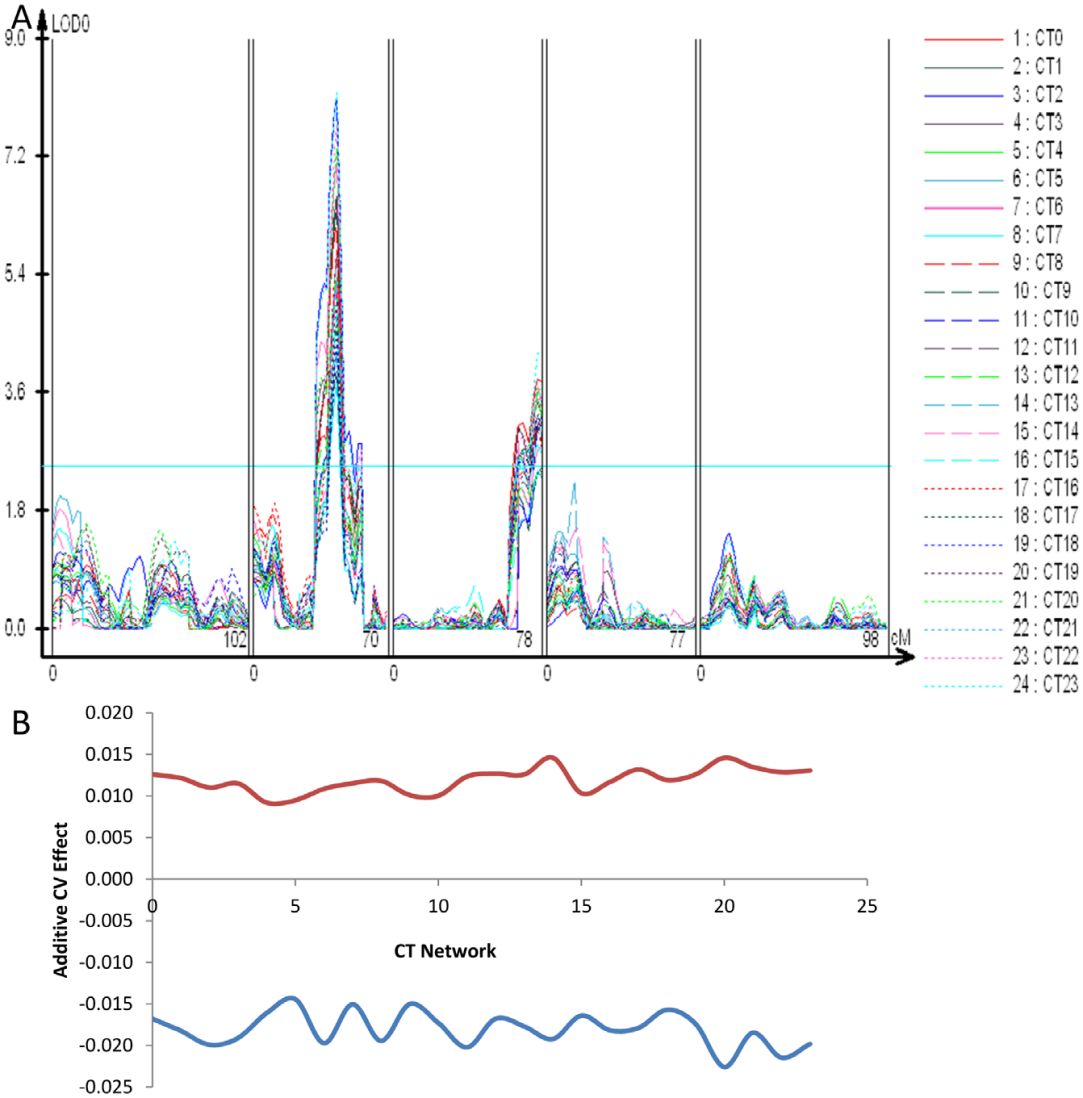
CT phase group CV QTL for circadian clock outputs. All transcripts previously identified as being regulated by the circadian clock were grouped into 24 different CT phase groups based upon the transcripts time of peak expression (CT) during the 24 hour photoperiod [82]–[84]. For instance, a transcript that peaks at CT 0 is binned within the CT0 phase group. All individual transcripts values within a CT phase group were then Z normalized and the average across all transcripts per CT phase group was obtained to derive a single expression estimate for each CT phase group for each microarray. These were then used to estimate the variance of the CT phase groups expression as described. (A) LOD plot for the 23 CT phase groups. (B) The additive effect of the Sha allele (y-axis) at the putative *ELF3* CV QTL (Blue) and the Chromosome III QTL located between *At3g58680* and *At3g61100 (Red)* is shown across all 24 CT phase groups (x-axis).

The chromosome II locus found using the global CVs of transcript abundance, glucosinolate accumulation and glucosinolate network expression maps close to the previously identified *ELF3* QTL (Figure 1, Figures S1 and S2) [81]. Allelic variation in *ELF3* between Bay and Sha has been shown to affect circadian rhythms and shade avoidance responses but not the wave form of the circadian oscillation [81]. We next wanted to test if the *ELF3* locus could be the same as the global CV eQTL hotspot. Because of *ELF3's* involvement in the circadian clock, we first asked whether we could identify stochastic noise QTL for circadian rhythms in the Bay x Sha population and whether these QTL would overlap the *ELF3* region. Circadian rhythms in transcript abundance have been measured in this population [82]; we used this same approach to map CV for the expression of circadian clock regulated genes. Briefly, transcripts previously identified as being regulated by the circadian clock were grouped into 24 CT phase groups based upon each transcript's time of peak expression (CT) during the 24 hour photoperiod [82]-[84]. Transcript expression values were then Z normalized and a single expression estimate was independently obtained for each CT phase group for each microarray. These were then used to estimate the variance of the CT phase groups expression as described. Both the *ELF3* locus and the chromosome III hotspot were found to alter CV for gene expression across the circadian clock output networks with opposing effects as had been found for general gene expression (Figure 5 and Figure 7). In contrast, the other identified *trans*-CV eQTL hotspots (Figure 5), do not appear to influence the CV of transcripts regulated by the circadian clock (Figure 7 versus Figure 5).

**Figure 8:**
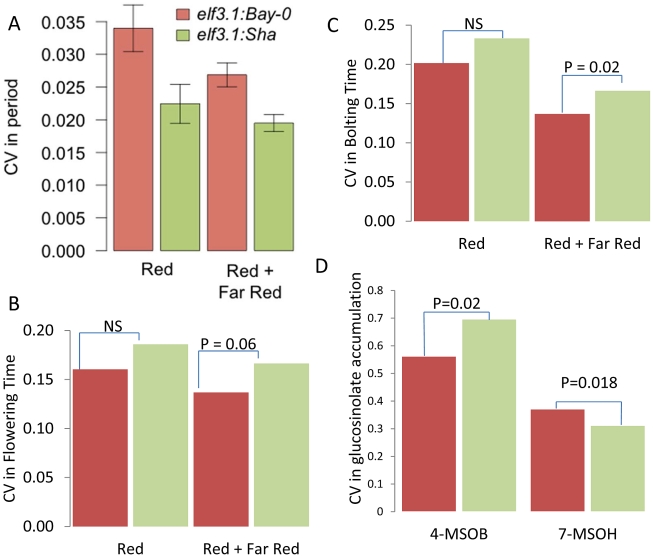
*ELF3* alters phenotypic CV. (A) Average coefficient of variance for the *elf3-1:ELF3Bay* and *elf3-1:ELF3Sha* T1 transgenic plants from 10 experiments performed in either constant red or red plus far red light. The mean coefficients of variance are statistically different between the alleles with a *p* = 0.0019 via ANOVA. Results were also significant by Levene's F-test. (B) Coefficient of variance for flowering time in the *elf3-1:ELF3Bay* and *elf3-1:ELF3Sha* T1 transgenic plants from 10 experiments performed in either constant red or red plus far red light. Significance by Levene's F-test is shown. (C) Coefficient of variance for the time to bolting in *elf3-1:ELF3Bay* and *elf3-1:ELF3Sha* T1 transgenic plants from 10 experiments performed in either constant red or red plus far red light. Significance by Levene's F-test is shown. (D) Coefficient of variance in 4-methylsulfinylbutyl (4-MSOB) and 7-methylsulfinylheptyl (7-MSOH) glucosinolate in *elf3-1:ELF3Bay* and *elf3-1:ELF3Sha* T1 transgenic plants from 9experiments. Significance by Levene's F-test is shown.

To test if *ELF3* is the causative gene controlling stochastic noise in this region we utilized previously generated Col-0 *elf3.1* knockout mutants lines containing a *CCR2:luc* reporter gene that were rescued with the genomic Bay and Sha *ELF3* alleles (*elf3:Bay-0* and *elf3:Sha*) [81]. Since Bay and Sha *ELF3* genomic alleles have been shown to affect the period of *CCR2:luc* oscillations in free running conditions under different light environments [81], we monitored the CV in period in at least 650 T1 plants per transgene distributed in 10 independent experiments performed in constant red or in constant red plus far red light. Independent of light conditions we found that the Sha *ELF3* allele reduced stochastic noise in the circadian oscillation period, in agreement with the direction of the global CV eQTL and circadian CT phase group QTL at the *ELF3* position (Figure 7 and Figure 8). Although plants in both red and red plus far red light presented lower CV ((*P* = 0.002 in red light versus *P* = 0.043 in red plus far red light, via ANOVA), the difference in CV between the two alleles was not significantly affected by the light treatment (Figure 8, *P* = 0.35 via ANOVA). The Bay and Sha alleles of *ELF3* did not affect CV for amplitude, phase or quality of the rhythms (measured as the relative amplitude error) in the transgenic plants (*P* = 0.10, *P* = 0.18 and *P* = 0.50 respectively, data not shown).

To further test if *ELF3*, could be the gene underlying other the CV QTL identified for other phenotypes at this locus, we tested if the transgenic lines differed in the level of stochastic noise for glucosinolate metabolites (Figure 1). Different alleles of *ELF3* led to changes in glucosinolate stochastic noise with the Sha *ELF3* allele increasing stochastic noise for the short chain aliphatic glucosinolate 4-methylsulfinylbutyl (4-MSOB) but decreasing stochastic noise for the long chain aliphatic glucosinolate 7-methylsulfinylheptyl (7-MSOH) (Figure 1 and Figure 8). Since the different alleles of *ELF3* (Bay v Sha) have also been shown to affect flowering time [81], we measured two traits related to this character in the transgenic lines and found that variation between the *ELF3* alleles led to differences in stochastic noise for flowering (Figure 8, Figure S4).

The observation that the Sha allele of *ELF3* led to higher stochastic noise in flowering time and 4-MSOB accumulation, whereas it was also associated with lower stochastic noise in circadian periodicity and 7-MSOH accumulation suggests that ELF3 is not simply making the plant more or less robust but instead is partitioning noise between specific phenotypes (Figure 8). Interestingly, this differential effect of *ELF3* upon stochastic noise agrees with the observed CV eQTL at this locus.: The Sha allele at the *ELF3* QTL was associated with decreased stochastic noise of transcriptional networks for circadian genes and most glucosinolate networks but the Sha allele had increased stochastic noise in the *FLC* (*At5G10140* –*Flowering locus C*) and *GS-OX2* (*At1g62540*) transcripts (Table S1) [85]–[88]. The increased noise in FLC nicely correlates with the observed flowering time noise. Furthermore, *GS-OX2* is required to synthesize 4-MSOB and concordantly links to the increased noise in this metabolite. Interestingly, *YUCCA3* (*At1g04610*) transcript accumulation also shows a CV eQTL at the *ELF3* locus suggesting a potential impact on auxin by this locus [89]. In summary, our results show that natural variation in *ELF3* leads to changes in stochastic noise in both plant and molecular phenotypes and that the direction of effect depends upon the specific phenotype. Therefore, *ELF3* is not a gene leading to plants displaying a general increase in phenotypic noise but instead affects noise in a network specific manner. Finally, it should be noted that there is no measurable difference in gene expression between the Bay and Sha alleles at *ELF3* showing that these altered stochastic noise phenotypes in metabolism, transcription and physiology are dependent upon the biochemical differences in the two alleles [40], [90].

## Discussion

The accurate measurement of any phenotype in biology produces two numbers, a measure of central tendency, such as the mean and a measure of variance. However, most genomic studies of quantitative genetics or systems biology in multi-cellular organisms limit the analysis to whether the genetic, environmental or developmental perturbation altered the phenotype's mean and typical do not analyze effects on the stochastic variance. However, numerous microbial and modeling analyses have shown that stochastic noise can be a meaningful phenotypic descriptor that contains information not conveyed by the average [21], [25], [26], [29], [30], [91]. We hypothesized that there may be an unrecognized and broad genomic distribution of natural variation in stochastic noise within higher eukaryotes. The data described in this report shows that there is significant genomic variation in phenotypic stochastic noise within the model plant, *Arabidopsis thaliana* within a single environment. This genetic variation in stochastic noise, as measured by CV, is highly heritable and influences multiple phenotypic levels ranging from transcripts to metabolites to complex physiology like circadian clock periodicity. We mapped numerous QTLs controlling metabolic and transcriptional CV and demonstrated that specific genes underlying these loci have the ability to influence the phenotypic CV for these traits. Further, phenotypes with higher stochastic noise had lower heritability. As such, it is likely that genetic variation in stochastic noise is widespread with a diverse mechanistic basis, and that to fully understand a quantitative trait both the mean and the stochastic variance of the phenotype need to be investigated.

Our QTL analysis showed that CV and average can be genetically separable measures of a phenotype. QTL mapping using phenotypic CV as the trait identified loci that were not found using the phenotypic average. One example is the transcriptome *trans*-CV eQTL hotspot on the bottom of chromosome III that did not appear as a major hotspot when using the average transcript accumulation to map eQTL (Figure 5) [40]. Further, natural variation at the *ELF3* locus impacts the average circadian clock period and flowering time while only effecting CV of the circadian clock (Figure 8 and Figure S4) [81]. Further, Levene's F-tests of the individual causal genes supports the use of CV to identify genes controlling natural variation in stochastic noise. Thus, directly interrogating stochastic noise as a separate measure of a phenotype can lead to new insights into the biology of a system.

### Extrinsic versus intrinsic noise

Stochastic noise is frequently divided into that which comes from sources internal to the organism (intrinsic) and environmental sources external to the organism (extrinsic) [26]. In unicellular organisms it is possible to use internal reporters and massive population sizes to begin to partition the two sources. This is much more difficult for multi-cellular organisms. However, in this study, a number of findings support that we are likely measuring largely intrinsic sources of noise rather than purely extrinsic sources. The first is that our measures of noise are correlated with the genotype of the organism, which would not have been true if the variance we were measuring was purely extrinsic/environmental noise. It could be argued that we are mapping genetic variation that leads to differential sensitivity to extrinsic noise. However, each experiment is highly replicated so to be mapping differential sensitivity to extrinsic noise would have required the sources of extrinsic noise to be the same in each experiment and to show similar variations across the experiments. While we can not entirely rule out this possibility, it is much more likely that we have identified loci controlling natural variation in intrinsic stochastic noise within a multi-cellular organism.

### Stochastic noise in plant/environment interactions and evolution

An interesting observation in this data is that there is an unexpectedly high genomic level of natural genetic variation controlling stochastic noise in transcripts, metabolites and physiology. The high frequency of *trans*-CV eQTL rules out the possibility that this is simply the mapping of large effect indel polymorphisms that would be expected to alter transcript CV in *cis*. Additionally, the finding that the genes underlying *trans-*CV eQTL also control stochastic noise in metabolites and complex physiology such as the circadian clock shows genetic control of stochastic noise impacts all levels of the plant. Interestingly previous reports have shown that HSP90 could be expected to control stochastic noise in numerous Arabidopsis phenotypes but we did not identify any trans-CV-eQTL hotspots linked to any of the known HSP90 genes [16]–[18], [92]. This suggests that natural variation in HSP90 is not a major driver of stochastic variation within this Arabidopsis population for this environment. It is possible that if we had used multiple environments that natural variation in HSP90 may have been identified but this was not the case.

Our findings raise the question of what genetically variable control of noise means in an ecological and evolutionary context. One possible answer would be that this genetic control of noise is meaningless because stochastic noise may not be under selection. However, this answer runs up against two impediments. The first is that in bacteria, natural variation in phenotypic stochastic noise has been shown to be adaptive under situations where the environment is highly unpredictable [25], [91], similar to that found in plant/herbivore interactions [28]. Additionally, several of the glucosinolate loci, including the *GSL.AOP2* locus that we show controls stochastic noise in glucosinolates, have been shown to be under selection in Arabidopsis and other related species [52], [56], [60], [93]–[100]. While these findings do not show that the stochastic noise variation is directly under selection pressure, it is clearly controlled by genetic loci that are themselves likely under selection pressure. Further, this suggests that selection is not solely focused upon decreasing stochastic noise within non-stressful environments especially for defense related traits.

The next question then becomes how natural variation in stochastic noise within environments that are not overtly stressful could benefit a multi-cellular organism. The answer to this might come in the form of a question that is related to the interest in identifying the genetic basis of local adaptation. However, the term local adaptation always engenders the response “what is local?”. It is possible that altering the stochastic noise of a system could alter the range of environments where it can successfully function. For instance, increasing the stochastic noise of the circadian period may enable that particular genotype to occupy more longitudinal niches, albeit at the likely cost of never being the optimal genotype in any specific niche. In contrast, decreasing the stochastic noise of the circadian period would optimize the fitness in a specific niche but likely at the loss of fitness across other niches. In this instance, natural variation in stochastic noise could lead to genetic control over what constitutes local for a specific genotype. As such, it may not be the variance itself that is adaptive, but instead the ability of variance to produce a more flexible network.

In contrast, stochastic noise in defense metabolites, such as glucosinolates, could represent a different benefit of natural variation in CV. Glucosinolates are a major anti-herbivore and anti-pathogen defense of Arabidopsis and relatives [53], [54], [58], [59], [98], [99] and as such could impart a pressure upon these herbivores and pathogens to counter adapt [101]. One mechanism that has been suggested as effective in slowing counter-adaptation is to increase the unpredictability of the defense compound (i.e. stochastic noise) [27]. As such, genetic control on the stochastic noise of defense compounds could in and of itself provide direct benefits to the efficaciousness of the defense. However, the observation that there is natural variation in stochastic noise of defense metabolites would suggest that high levels of noise are not always beneficial, possibly depending upon the ratio of generalist and specialist herbivores in a given genotype's normal locale [98]. Testing these different potential benefits of stochastic noise will require the development of genotypes that differ solely in stochastic noise to allow this effect to be partitioned away from any influence upon the mean phenotype.

### Genetic control of stochastic noise and systems biology

A major difficulty in systems biology is the presence of massive datasets that are largely correlative when comparing different transcripts. This has lead to numerous attempts to derive causal information from these correlative datasets. However, even the best approaches are susceptible to a number of systemic errors that deal with predicting regulatory loop structure as well as combinatorial regulation [102]. For regulatory loops, correlative approaches using average responses generate a number of possible network topologies that are similar with respect to regulation of phenotypic average, but that make very different predictions about how perturbations will control the stochastic noise of the system [5], [6], [103]–[105]. Given this, it may be possible to use the presence of genetically controlled stochastic noise to help better refine systems biology models. The mean transcript, protein, or metabolite levels could be used to generate multiple initial models that could then be analyzed by using the stochastic noise in the system to determine which model most accurately predicts the observed stochastic noise. Future work on this approach could be useful but would require true independent replication in systems biology experiments to allow accurate estimations of stochastic noise for each measured phenotype.

### Future potential

The identification that stochastic noise of phenotypes has a level of genetic control that appears to be on par with that observed for the phenotype average suggests that there is a fount of phenotypic information that has largely not been studied in most modern genetic, genomic or systems biology studies. For instance, numerous natural and induced mutant screens and surveys have been conducted in Arabidopsis to determine the genes controlling the phenotypic average [106]–[108]. Similar large scale approaches have been conducted in numerous other organisms focused on phenotypic averages [109]–[111]. While these have provided great advances in our understanding of biology, it raises the question of what would happen if we repeat these screens and surveys to identify genetic variation controlling stochastic noise in phenotypes. Would we identify the same genes or would we begin to identify a large suite of previously unknown genes that control stochastic variation rather than phenotypic average? Experiments focused on the stochastic nature of a phenotype require independent replication but could yield a new view of organismal biology that is currently specified by our focus upon phenotypic averages.

## Materials and Methods

### Measuring transcript CV

To directly estimate the CV for each individual genes transcript accumulation (22,746 transcripts in total) as a separate phenotype within the Bay x Sha RIL population [42], we obtained two independent microarray experiments (TABM-224 and TABM-518) wherein 211 RILs were each measured in duplicate within each experiment providing four replications [40]. Raw image data from the RIL GeneChips were converted to numeric data via Bioconductor software (www.bioconductor.org). We utilized quantile normalization across all arrays to reduce non-biological variation coming from the technology itself, and when applied at the probe level it has been shown to outperform other normalization methods that are based on what is referred to as a “base-line array” [112]. After quantile normalization, we utilized the absolute expression values to measure the CV for each gene separately for each experiment using σ/µ [21], [26], [113], thus providing two independent biological replicate measures of CV for each gene. The use of CV as a direct phenotype has previously been used in a number of instances. By measuring the within line CV as a phenotype for the Bay x Sha population allows us to then utilize CV as a direct measurement of stochastic variation as a phenotype. The level of per line replication for the array data does not support the use of Levene's variance tests or measures. Additionally, all lines were planted and harvested within a randomized complete block design at all stages thus limiting any potential technical bias to generate these observations [40], [77].

### Measuring network expression CV

To estimate the CV for specific transcript networks, we utilized a previously published approach whereby we average the expression across a group of genes to provide an estimate of the gene network's expression value [38], [114]. Briefly, this network approach uses any *a priori* defined group of genes as a network. Every transcript that is defined within a network is z transformed to place them all on the same scale. For every microarray within the dataset, the network expression value is obtained by averaging across the z values for all transcripts within the network. This provides a single network value that can then be utilized for downstream applications. This approach has previously been used to map network QTL controlling the difference in average expression [63] and can be extended to identify differences in network stochasticity using the CV value instead of the average expression. Gene membership within specific circadian networks were defined as previously described [83]. Gene membership within glucosinolate pathways were defined as previously described [63], [67]. This approach was also used to generate a global CV average by averaging the CV across all 22,746 transcripts measured on the ATH1 Affymetrix microarray.

### Measuring glucosinolate metabolite CV

To estimate the CV for specific defense metabolites, we utilized previously published data wherein the µ and σ for a large set of glucosinolates within a Bay x Sha RIL population consisting of 403 lines had been measured [63]. The glucosinolates were measured in a similar growth stage and growth chamber as that for the transcriptomics analysis allowing for better comparison between the datasets [40], [63]. For measuring altered glucosinolate metabolite CV in the independent transgenic lines, we compiled data from multiple independent experiments that had previously been published in separate papers. We analyzed the same lines in at least four independent experiments with replication allowing us to test if the CV differed across these genotypes [58], [63], [67], [73].

Glucosinolate genotype analysis: To test if variation at specific glucosinolate genes could alter the CV of either metabolite or transcripts, we obtained previously published data involving multiple independent biological replicates for the following genotypes all of which are generated within the Arabidopsis Col-0 accession background. To elevate MYB gene expression, we used previous lines where the Arabidopsis Col-0 versions of *MYB28*, *29* and *76* were separately introduced back into Arabidopsis Col-0 using a 35S promoter to induce their expression – *35S:MYB28*, *35S:MYB29* or *35S:MYB76* [73]. To mimic natural variants that have low to no expression of *MYB28*, *29* or *76*, we used previously obtained insertional T-DNA mutants within each of these genes obtained from the Arabidopsis Col-0 accession; *myb28-1*, *myb29-2* and *myb76-1* [67], [73]. All insertional T-DNA mutants underwent at least one backcross and had previously been shown to abolish or dramatically diminish MYB gene expression [67], [73]. To mimic the natural variation at the *AOP2* locus, we utilized the Arabidopsis Col-0 accessions that contains a natural knockout of *AOP2* and introduced the functional enzyme encoding gene back into this natural null background [63], [115]. Thus, all of these lines are single gene manipulations of major glucosinolate loci within a common genomic background, Col-0.

### Estimation of CV heritability

For estimating broad-sense heritability, we utilized the independent measures of CV directly as a phenotypic measure. This allowed us to estimate broad-sense heritability (H) for each CV phenotype as H = σ^2^_g_/σ^2^_p_, where σ^2^_g_ is the estimated CV phenotypes genetic variance among different genotypes in this sample of 211 RILs, and σ^2^_p_ is the CV phenotypic variance for each phenotype [116]. Heritability was estimated for all expression phenotypes. The metabolite phenotypes did not have the individual values from each independent experiment, and therefore, heritability was not measurable.

### Mapping QTL for CV phenotypes

To map QTL for the CV phenotypes, metabolic, network and individual gene expression, we measured the average CV for each phenotype across all experiments and used the average CV in conjunction with a previously generated map for 211 Bay x Sha RILs ([42], [77]; see also the file “Average CV per transcript per RIL” at http://plantsciences.ucdavis.edu/kliebenstein/TableS1Plosgenetics.txt [note: this file is ∼28 MB]). For glucosinolates, we utilized a larger collection of 400 Bay x Sha RILs [42]. Composite interval mapping (CIM) analysis [117] was employed in conjunction with the 5 cM framework map. The “*zmapqtl*” CIM module of QTL-Cartographer Version 1.17 [118] with a walking speed of 1 cM and a window size of 10 cM was employed to analyze each phenotype. To obtain a threshold criterion for declaring statistically significant eQTL, a global permutation threshold was obtained by permuting the e-traits while maintaining the genetic information [40]. For each of 100 randomly selected phenotypes, the null distribution of the maximum likelihood ratio test (LRT) statistic was empirically estimated using permutation thresholds based on 1,000 permutations [40], [78]–[80]. We then utilized the 95th percentile permutation threshold across the 100 null distributions [40]. We utilized the resulting output to localize, summarize and count CV QTL using the Eqtl module of QTL-Cartographer in conjunction with the previously optimized 5 cM exclusionary window where no CV QTL can be closer than 5 cM to the nearest QTL (Table S1) [40], [118]. This is an identical approach at all stages to that used to previously map the eQTL for this dataset and as such should increase the direct comparison between datasets [40]. Additionally, we have been able to clone and biologically validate causal loci controlling several of the trans effect loci controlling subtle shifts in physiological networks as identified from the eQTL analysis [90], [119].

We have previously shown that single-feature polymorphisms are not a significant difficulty in this population for this array data when estimating expression values [40], [77]. As such, we did not control for potential single-feature polymorphism issues. The low level of cis-CV-eQTL within our results further supports this observation.

### CV QTL hotspot significance threshold

To determine whether a genetic location associated with multiple CV QTLs was a significant cluster or ‘hotspot’, we estimated a significance threshold using permutation as previously described for transcriptomic data [40], [90], [109], [120]–[123]. The positions of the 98,014 CV eQTLs (Table S1) at the marker intervals were permuted across the genome 1,000 times, and the maximal number of CV eQTLs per genetic position per permutation was obtained. Using the distribution of the maximum number of CV eQTLs, the criterion for declaration of a significant eQTL hotspot was 422 CV eQTLs per genetic position at alpha =  0.05. The permutated hotspot approach has been used to identify genes that cause the transcriptional difference for a number of hotspots showing that this approach is identifying biologically validatable effects [39], [63], [120], [124], [125].

### ELF3 transgenic plants

*elf3-1* null mutants carrying the *CCR2::luc* reporter gene were obtained from Dr. Stacey Harmer (University of California, Davis). Full genomic clones of *ELF3* from Bay and Sha including 1.5 kb of upstream promoter were cloned in pJIHOON212. *elf3-1-CCR2::luc* plants were transformed with these constructs using *Agrobacterium tumefaciens* [81], [126]. To account for differences between *elf3.1: Bay* and *elf3.1:Sha* due to the transformation protocol, transgenic plants obtained from two independent batches were used, but no effect of the *Agrobacterium* inoculate was detected (*P* = 0.31 via ANOVA, data not shown).

### Measuring CV of circadian period for *ELF3* alleles

*elf3:Bay* and *elf3:Sha* transgenic T1 seeds from two different Agrobacterium transformation batches were placed on MS medium with the appropriate antibiotic and stratified for 4 days (4°C, dark). After entrainment under white light in 12∶12 photoperiods for 7 days, resistant plants were transferred to new MS plates and moved to continuous red light or red + far-red light conditions, where luminescence was recorded for 6 to 7 days.

Five independent experiments were conducted in continuous red light (R, total PAR of 64 uE) and 5 experiments in continuous red plus far red light (R+FR, total PAR of 64uE with a R:FR ratio of 0.5) conditions created with LED lights. Plants were monitored using a CCD camera taking pictures every 2 hours. The data collected was analyzed for rhythmicity using the luciferase activity method described in [127]. Only plants showing stable rhythms (Relative Amplitude Error below 0.5) were considered for the analysis. Between 12 and 150 T1 plants (average 75.2, median 86) for each transgene were included in each experiment. Coefficient of variance was calculated as the standard deviation divided by the mean period estimate for each transgenic line in each experiment.

### Measuring variance in glucosinolate accumulation between *ELF3* alleles

*elf3:Bay* and *elf3:Sha* transgenic T1 seeds from three different *Agrobacterium tumefaciens* transformation batches were planted on soil including *elf3.1* mutants and WT Col-0 as a control. The extreme hypocotyl length, flowering time and cotyledon color phenotypes of the *elf3.1* mutants were assessed to distinguish transformed from untransformed plants [128]. Transformed plants were grown for 25 days in a 10 hour photoperiod. At 25 days, leaf tissue was harvested from each plant and individually extracted and assayed via HPLC for glucosinolate identity and concentration as previously described [72], [115]. The experiment was replicated 9 times for a total of 106 *elf3:Bay* and 108 *elf3:Sha* independent T1 plants. Levene's F-tests were used to compare variance between the two T1 genotype classes.

## Supporting Information

Figure S1 Individual trait CV eQTL for Aliphatic GLS biosynthetic network. CV eQTL were mapped using the expression levels of 60 transcripts associated with *Arabidopsis thaliana* glucosinolate biosynthesis using the four replicate microarrays for the Bay x Sha population. The transcripts controlled by the most significant CV eQTL are labeled. The AOP2 and AOP3 labels are in the position of the genes and are cis CV eQTL. The top panel shows LOD score and the bottom panel shows the additive effect. The *GSL.MAM* and *GSL.MYB2976* loci are labeled for reference.
(TIF)

Figure S2 Pathway CV QTL for GLS related biosynthetic networks. QTL analysis of pathway CV QTL across the five Arabidopsis chromosomes for 13 different metabolic pathways associated with glucosinolate accumulation. All transcripts associated with 13 different metabolic pathways were compiled to estimate the average and standard deviation of pathway expression per line across all the transcripts in the pathway. This was then used to estimate the pathway CV per line and this was utilized to map QTLs for each network as described. The position of the *GSL.AOP*, *MYB2976* and *MAM* loci are shown with respect to the x-axis. A. LOD value for pathway CV QTL. Line color legend is shown in B. B. QTL locations for pathway CV QTL. The vertical line within each bar shows the statistical peak and the bar shows the region of significance for each QTL. C. Graph of estimated additive effects for pathway CV QTL based upon the Sha allele.
(TIF)

Figure S3 Distribution of CV eQTL per transcript. The number of CV eQTL per transcript across all 22,746 transcripts CV's per RIL.
(TIF)

Figure S4 *ELF3* HIF alters Flowering Time CV. Average coefficient of variance of HIF M for Flowering time in either constant red (Shade) or red plus far red light (Sun). The mean coefficients of variance were tested for significant differences using a paired Levene's F-test and the P values are shown.
(TIF)

Table S1 List of CV eQTL for transcriptomics analysis of Bay x Sha.
(XLSX)
