# Supplementary figures and images for: Genomic Analysis of QTLs and Genes Altering Natural Variation in Stochastic Noise
Source: PLoS Genet. 2011 Sep 29;7(9):e1002295. doi: 10.1371/journal.pgen.1002295 (PMC3183082; doi:10.1371/journal.pgen.1002295)

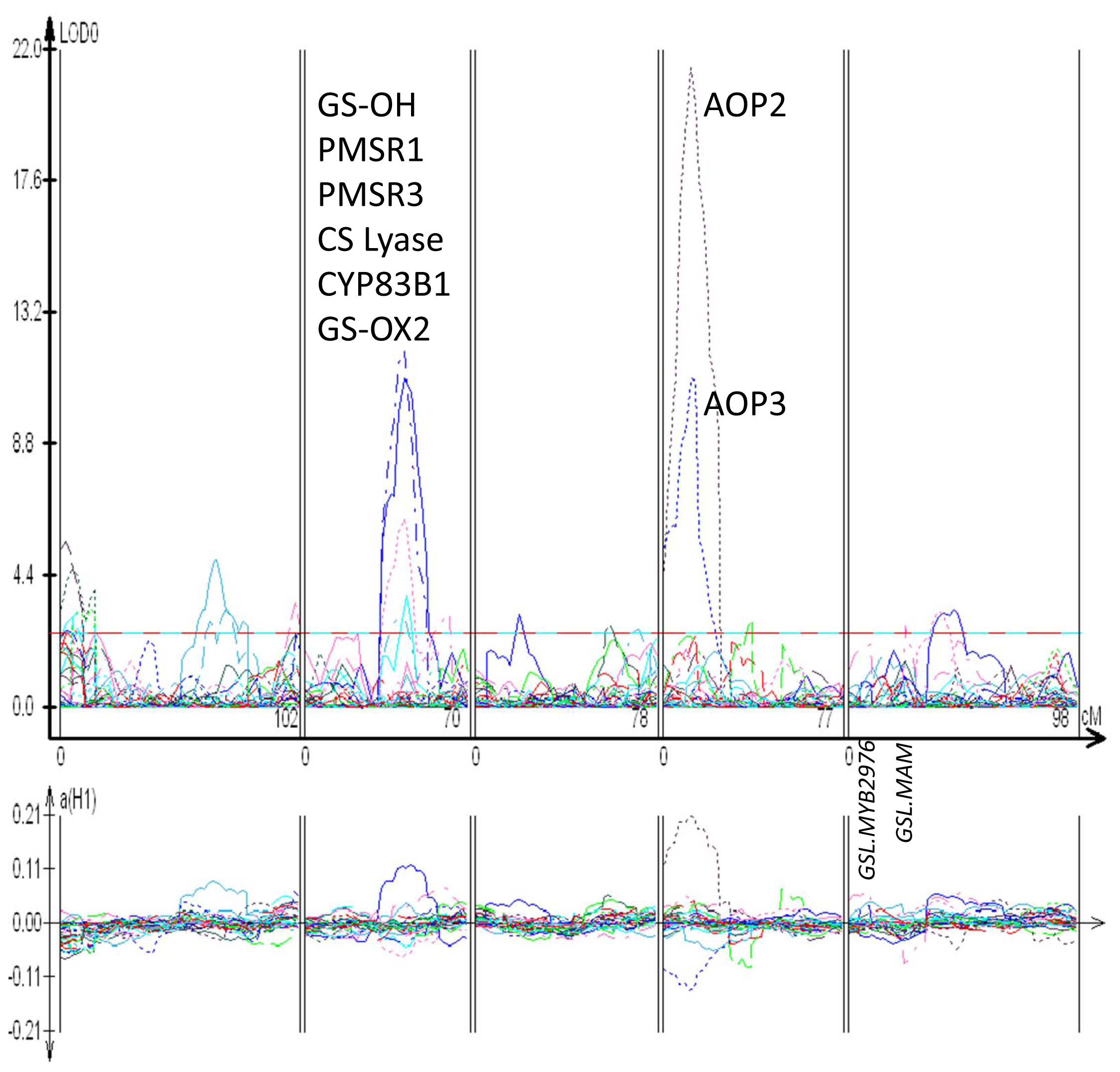

Supplement: Figure S1 — Individual trait CV eQTL for Aliphatic GLS biosynthetic network. CV eQTL were mapped using the expression levels of 60 transcripts associated with Arabidopsis thaliana glucosinolate biosynthesis using the four replicate microarrays for the Bay x Sha population. The transcripts controlled by the most significant CV eQTL are labeled. The AOP2 and AOP3 labels are in the position of the genes and are cis CV eQTL. The top panel shows LOD score and the bottom panel shows the additive effect. The GSL.MAM and GSL.MYB2976 loci are labeled for reference. (TIF) [file pgen.1002295.s001.tif]

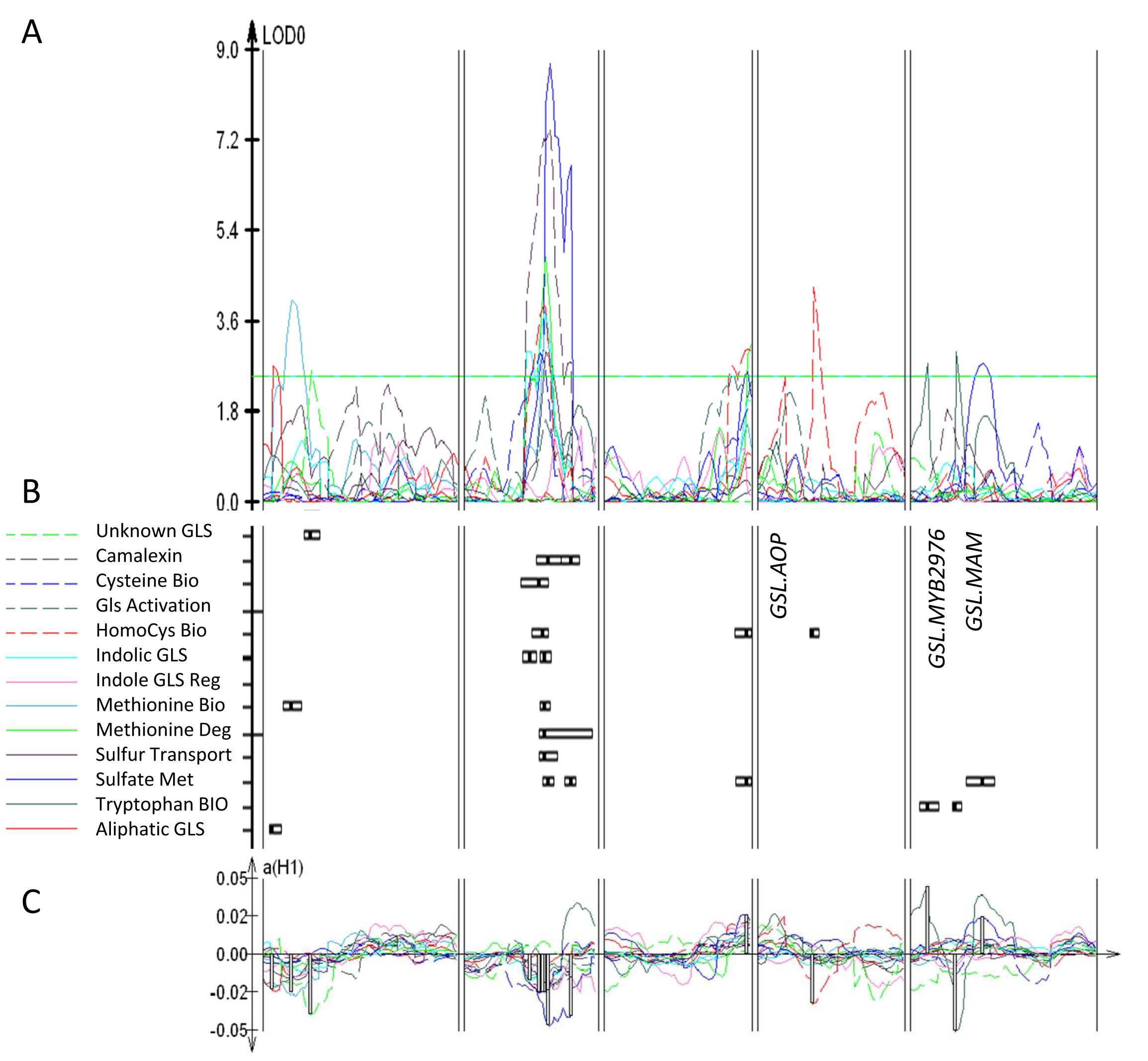

Supplement: Figure S2 — Pathway CV QTL for GLS related biosynthetic networks. QTL analysis of pathway CV QTL across the five Arabidopsis chromosomes for 13 different metabolic pathways associated with glucosinolate accumulation. All transcripts associated with 13 different metabolic pathways were compiled to estimate the average and standard deviation of pathway expression per line across all the transcripts in the pathway. This was then used to estimate the pathway CV per line and this was utilized to map QTLs for each network as described. The position of the GSL.AOP, MYB2976 and MAM loci are shown with respect to the x-axis. A. LOD value for pathway CV QTL. Line color legend is shown in B. B. QTL locations for pathway CV QTL. The vertical line within each bar shows the statistical peak and the bar shows the region of significance for each QTL. C. Graph of estimated additive effects for pathway CV QTL based upon the Sha allele. (TIF) [file pgen.1002295.s002.tif]

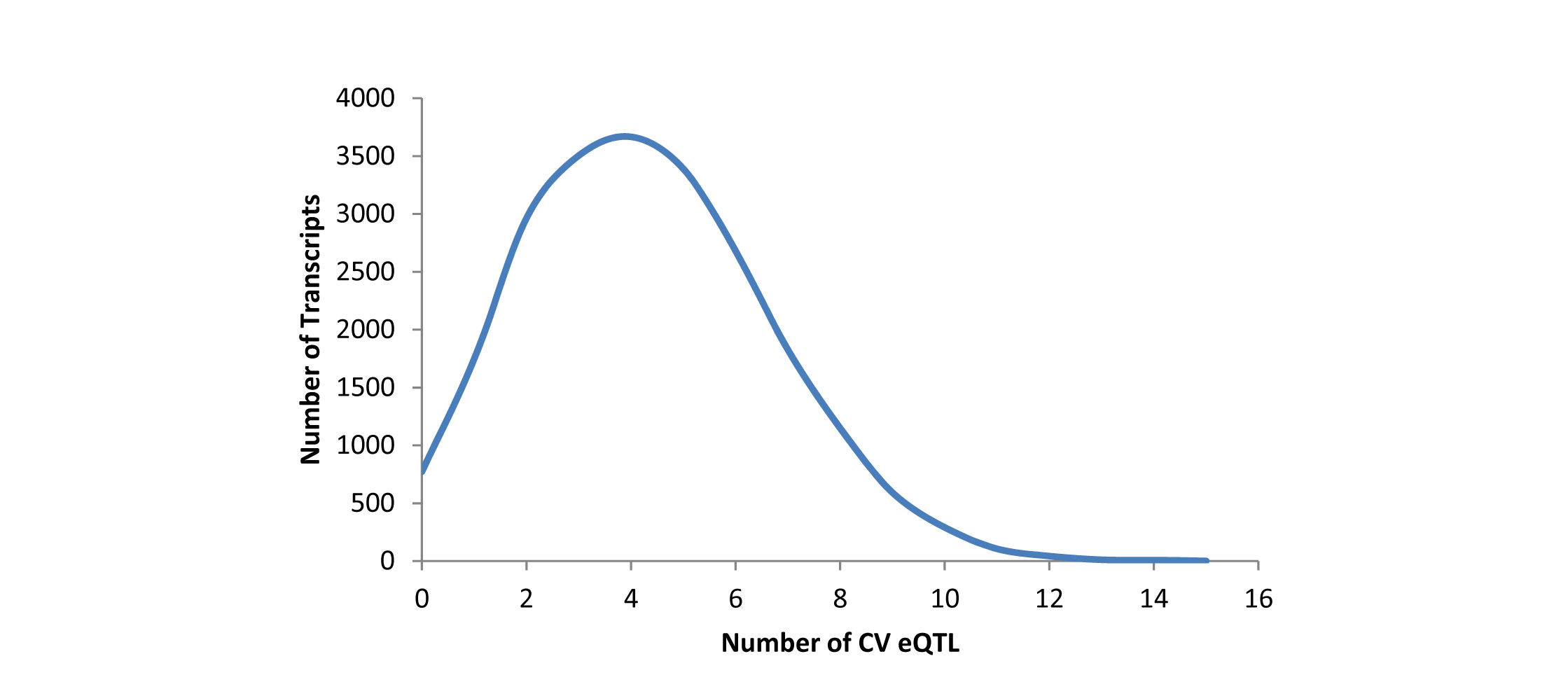

Supplement: Figure S3 — Distribution of CV eQTL per transcript. The number of CV eQTL per transcript across all 22,746 transcripts CV's per RIL. (TIF) [file pgen.1002295.s003.tif]

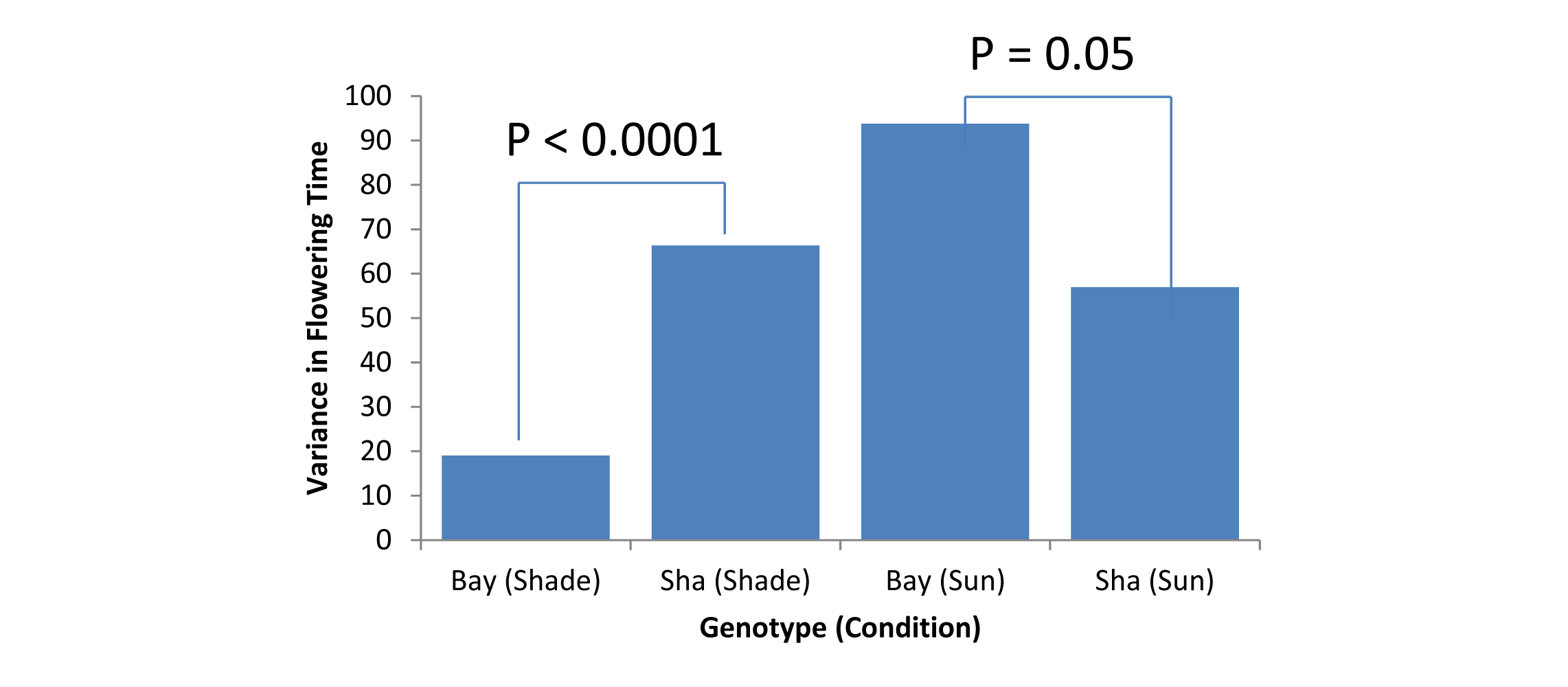

Supplement: Figure S4 — ELF3 HIF alters Flowering Time CV. Average coefficient of variance of HIF M for Flowering time in either constant red (Shade) or red plus far red light (Sun). The mean coefficients of variance were tested for significant differences using a paired Levene's F-test and the P values are shown. (TIF) [file pgen.1002295.s004.tif]
